# A randomized controlled trial on errorless learning in goal management training: study rationale and protocol

**DOI:** 10.1186/1471-2377-13-64

**Published:** 2013-06-20

**Authors:** Dirk Bertens, Luciano Fasotti, Danielle HE Boelen, Roy PC Kessels

**Affiliations:** 1 Donders Institute for Brain, Cognition and Behaviour, Radboud University Nijmegen, Nijmegen, The Netherlands; 2 Rehabilitation Medical Centre Groot Klimmendaal/SIZA Support and Rehabilitation, Arnhem, The Netherlands; 3 Department of Medical Psychology, Radboud University Nijmegen Medical Centre, Nijmegen, The Netherlands

**Keywords:** Goal management training, Errorless learning, Executive deficits, Acquired brain injury

## Abstract

**Background:**

Many brain-injured patients referred for outpatient rehabilitation have executive deficits, notably difficulties with planning, problem-solving and goal directed behaviour. Goal Management Training (GMT) has proven to be an efficacious cognitive treatment for these problems. GMT entails learning and applying an algorithm, in which daily tasks are subdivided into multiple steps. Main aim of the present study is to examine whether using an errorless learning approach (preventing the occurrence of errors during the acquisition phase of learning) contributes to the efficacy of Goal Management Training in the performance of complex daily tasks.

**Methods/Design:**

The study is a double blind randomized controlled trial, in which the efficacy of Goal Management Training with an errorless learning approach will be compared with conventional Goal Management Training, based on trial and error learning. In both conditions 32 patients with acquired brain injury of mixed etiology will be examined. Main outcome measure will be the performance on two individually chosen everyday-tasks before and after treatment, using a standardized observation scale and goal attainment scaling.

**Discussion:**

This is the first study that introduces errorless learning in Goal Management Training. It is expected that the GMT-errorless learning approach will improve the execution of complex daily tasks in brain-injured patients with executive deficits. The study can contribute to a better treatment of executive deficits in cognitive rehabilitation.

**Trial registration:**

(Dutch Trial Register): http://NTR3567

## Background

Brain-injured patients referred for outpatient rehabilitation frequently experience difficulties with planning, problem solving, reasoning and goal directed behaviour [1-3]. These difficulties can be characterized as executive deficits [1,4-6] and compromise daily functioning and even functional independence [7,8]. More specifically, dysfunction of these higher-level control processes leads to real-life everyday disorganization [9] and even subtle executive deficits often provoke difficulties in the performance of everyday-life tasks [10]. Because of the high prevalence of executive dysfunction in the brain-injured population [11] and its considerable impact on everyday life, effective treatment is warranted.

### Goal management training

**Figure 1:**
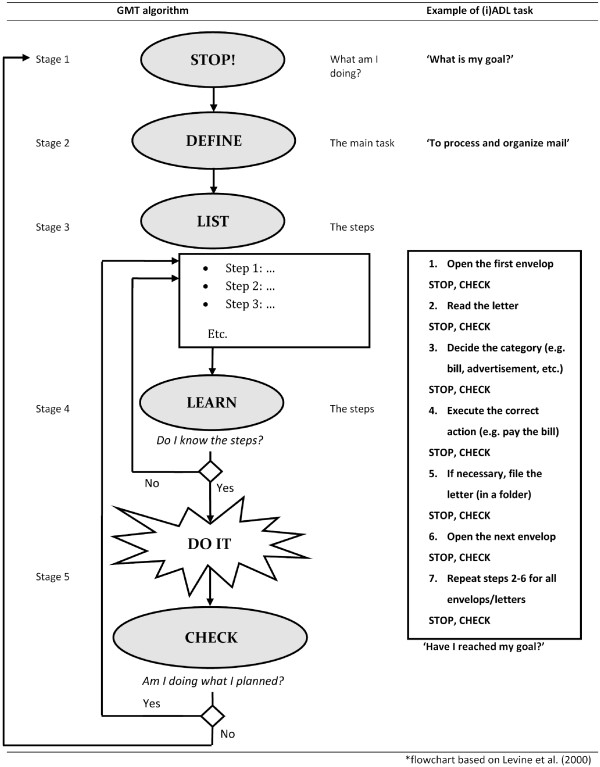
**Flowchart of the GMT algorithm and an example of its application adapted from [**[20]**].**

Based on Duncan’s [12] theory of goal neglect, Robertson [13] developed Goal Management Training (GMT). GMT is a rehabilitation technique aimed at helping patients with executive impairments to better structure (instrumental) activities of daily living ((i)ADL). GMT entails learning and applying an algorithm, in which complex tasks are subdivided into multiple task steps. Both, the final goal and the task steps leading to this goal have to be kept active in working memory. Unfortunately, working memory processes are often impaired in patients with executive deficits. Monitoring goal-directed behaviour and the correct execution of task steps are the main aims of GMT. GMT can be applied to (re)learn all sorts of (i)ADL tasks, for example cleaning up the living room, processing and organizing mail or making a day schedule. In Figure 1 both the GMT algorithm and an example of its application are illustrated. Previous studies have established the efficacy of GMT [7,11,14-17] and the training is widely applied in the field of cognitive rehabilitation. The acquisition of the algorithm and the performance of the task steps, however, relies on self-control, which is impaired in patients with executive problems [6,18]. Consequently, errors that occur during the acquisition of the algorithm and the learning of the task steps are not corrected and may interfere with the correct acquisition of the GMT process and the correct performance of the task [19]. Preventing the occurrence of errors during learning, also known as errorless learning, may enhance treatment effects.

### Errorless learning in goal management training

In errorless learning, the occurrence of errors during the learning phase is prevented in contrast to standard learning, or trial and error learning, in which errors may occur naturally. Fillingham et al. [20] described the mechanism of errorless learning using the Hebbian learning model [21]. Learning is described as a strenghtening of the connection between neurons that fire together. If a stimulus is followed by a reponse, the subsequent pattern of neural activitivity will be more likely to be activated again in similar situations. This means that the same response can be expected, even if it is an incorrect action [22]. If an errorless learning approach is applied in this process, the activation of incorrect neural patterns will be prevented and erroneous actions will not be evoked.

In clinical practice, several errorless learning techniques can be applied during training of complex daily tasks. Task steps can be taught using cue cards, (feed-forward) verbal instructions and visual demonstration or modeling by the trainer [23-25]. Several studies have shown that the quality of task performance after errorless learning is superior compared to errorful learning in patients with cognitive impairments of different aetiologies [26-32]. Most studies on errorless learning have focussed on patients with memory deficits. In these studies the efficacy of errorless learning is explained by the mechanism that errors are not consciously corrected because of impairments in explicit memory, but implicitly consolidated through a relatively intact implicit memory system [33-35]. However, other studies do not agree with this hypothesis and describe the benefits of errorless learning by residual explicit memory processes [36,37]. Another mechanism that may explain the advantage of errorless learning in patients with executive disorders is that errors are not detected due to a failing error-monitoring system [38,39] and the inability to adjust behaviour on the basis of feedback [26]. By preventing the occurrence of errors in learning the execution of a task, both these systems are circumvented.

The main aim of the current study is to examine the efficacy of Goal Management Training using an errorless learning approach in the treatment of executive impairments in patients with acquired brain injury, focusing on (instrumental) activities of daily living ((i)ADL). Both GMT and errorless learning are two well investigated instructional methods of proven effectiveness. However, to date they have never been combined. Using an errorless learning approach in GMT may optimize both the acquisition of the GMT algorithm and the execution of complex tasks in daily living. To examine the efficacy of these combined techniques, (i)ADL task performance will be evaluated using a standardized observation scale taking correct, ineffective and missing steps into account [27]. The primary hypothesis is that combining errorless learning and GMT will result in a more efficacious intervention, when applied to (re)learning daily tasks in patients with executive disorders after acquired brain injury. This study may contribute to a better treatment of disorganized behaviour after brain injury and improve the cognitive rehabilitation of patients with executive disorders. From a patient perspective, it might consistently contribute to enhance the functional independence of brain-injured patients.

## Methods/Design

To evaluate the efficacy of GMT in which errorless learning is integrated, this approach will be compared with conventional GMT treatment in which an errorful approach is used. This comparison will be investigated in a double blind randomized controlled trial that is registered at the Dutch Trial Register (No. NTR3567). The Medical Review Ethics Committee region Arnhem-Nijmegen approved the study (No. NL38019.091.11).

### Participants and setting

The study population consists of brain-damaged patients referred for outpatient cognitive rehabilitation. Participants eligible for the study must have executive disorders due to acquired brain injury (ABI) of non-progressive nature (i.e. traumatic brain injury, stroke) in the chronic phase of the illness. Executive deficits will be assessed by an extensive neuropsychological examination.

#### Inclusion criteria

1. Non-progressive acquired brain injury;

2. Minimal post-onset time of 3 months;

3. Being in outpatient rehabilitation;

4. Having executive deficits, as established by neuropsychological examination;

5. Living independently at home;

6. Age: 18–70 years at onset.

#### Exclusion criteria

1. Inability to speak/understand the Dutch language;

2. Severe premorbid psychiatric problems;

3. Neurodegenerative disorders;

4. Substance abuse;

5. Severe cognitive comorbidity.

#### Setting

Patients will be recruited from the Rehabilitation Medical Centre Groot Klimmendaal in Arnhem, the Netherlands and the outpatient rehabilitation clinic for brain injured patients and the department of Neurorehabilitation of the Sint Maartenskliniek in Nijmegen, the Netherlands. In the course of 18 months 64 participants will be recruited.

### Procedure

**Figure 2:**
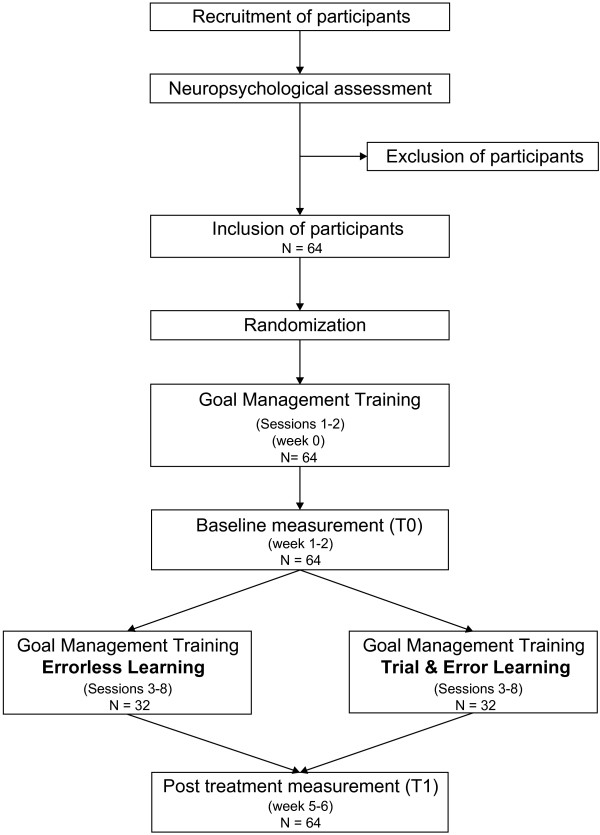
Flowchart of the study design.

A flowchart of the study design is presented in Figure 2. An extensive neuropsychological assessment will be performed as part of the selection procedure. Participants are eligible for the study if they have executive impairments, objectified by neuropsychological examination. As executive functioning is a multifarious concept, the neuropsychological assessment is designed to cover five of its main aspects. To assess response generation [2] the Category Fluency test (CFT) and the Letter Fluency test (LFT) will be administered. Planning will be measured with an altered version of the Modified Six Elements Test and the Zoo Map test (subtest of BADS) [40]. The Go/No-go task from the computerized TAP 2.1 [41] will be used to examine response inhibition. Working memory will be assessed with Letter-Number Sequencing (LNS; subtest of the WAIS III) [42] and task switching with the Brixton Spatial Anticipation test [43]. Specifically, the criteria for having executive disorders and to be included in the study are either a standard score of 1.5 standard deviation (SD) below the normative mean on at least two of the seven executive tests or a standard score between 1 and 1.5 SD below the normative mean on at least four of those seven tests. Moreover, to obtain a complete cognitive profile of the participants, the Rivermead Behavioural Memory Test-Third Edition (RBMT III) [44] will be administered to assess episodic memory and the alertness subtest of the TAP 2.1 [41] will be used as a measure for attention and concentration. The National Adult Reading Test (Dutch version) (NART) will be given to estimate premorbid IQ. All neuropsychological tests will be administered by a neuropsychologist or a trained assistant.

After fulfilling the inclusion criteria and obtaining the signed informed consents, participants will be randomly assigned to GMT with an errorless learning approach (experimental treatment) or to conventional GMT (treatment as usual) using trial and error learning.

### Interventions

Both treatment arms will comprise eight one-hour individual sessions, administered twice a week by trained therapists. An overview of the content of the training sessions is shown in Table 1. Sessions 1–4 will take place at the participating centers. Sessions 5–8 will take place at the participants’ home or in the work environment, depending on the treatment goals. The first two sessions of GMT will be identical for both conditions. In the first session participants will be informed about GMT in general and about cognitive dysfunction, more specifically about executive dysfunction after acquired brain injury. In the second session each participant will choose two individual treatment goals. These treatment goals must be (i)ADL tasks and the participant has to experience difficulties performing the chosen tasks. Learning the correct execution of these (i)ADL tasks will be the main aim during the rest of the training sessions. Acceptable treatment goals are those which can be subdivided into multiple steps and should be defined in accordance with the SMART method (Specific, Measurable, Attainable, Reasonable, Timely) [45]. After the second session a baseline assessment will take place. The execution of both treatment goals will be filmed, so that it can be evaluated later by assessors who are blind for allocation. After the baseline assessment participants will undergo training sessions 3–8 in the errorless learning condition or in the trial and error condition.

**Table 1:** Content of GMT sessions for both treatment arms

**Session**	**Contents**	
1	Providing information about training;	
	Providing information about cognitive functioning after acquired brain injury in general with an emphasis on executive impairments;	
	Increasing awareness of individual executive dysfunction;	
	Preparing the setting of two individual treatment goals	
2	Setting two individual chosen treatment goals (i)ADL tasks);	
	Filling out the Goal Attainment Scale forms for each treatment goal	
	**GMT-errorless learning**	**Conventional GMT-treatment**
3	Defining steps concerning treatment goal 1 using cue cards and other errorless learning techniques	General information about GMT and the GMT algorithm
4	Defining steps concerning treatment goal 2 using cue cards and other errorless learning techniques	Filling out GMT scheme by participant concerning treatment goal 1
5	Performance of the steps concerning treatment goal 1 using errorless learning techniques	Filling out GMT scheme by participant concerning treatment goal 2
6	Performance of the steps concerning treatment goal 2 using errorless learning techniques	Performance of treatment goal 1 according to the GMT algorithm
7	Integrating the steps of treatment goal 1 into the GMT algorithm using errorless learning techniques;	Performance of treatment goal 2 according to the GMT algorithm
	Performance of treatment goal 1 according to the GMT algorithm using errorless learning techniques	
8	Integrating the steps of treatment goal 2 into the GMT algorithm using errorless learning techniques;	Improvement of GMT schemes concerning one or both of the treatment goals and/or practicing performance of one or both of the treatment goals
	Performance of treatment goal 2 according to the GMT algorithm using errorless learning techniques	

Note. Sessions 1 and 2 are the same for both conditions.

#### GMT-errorless learning condition

The experimental treatment consists of GMT with an errorless learning approach, i.e., both the acquisition and application of GMT will be taught using error controlling methods. This implies active guidance from a therapist to prevent the occurrence of errors or guessing. Therefore, errorless learning techniques, such as verbal instructions, modeling and cue cards will be used, as well as written instructions of the chosen (i)ADL tasks. After the two tasks have been subdivided into multiple steps and have been rehearsed verbally during sessions 3 and 4, the actual execution of these steps is practiced in sessions 5 and 6 of the treatment. In these sessions cues will be faded after successful execution of the steps (i.e., without hesitation or errors). In sessions 7 and 8 the patient will be taught to check after each task step if the action was performed correctly and if it has led to the planned (subordinate) goals. ‘Checking’ is part of the final stage of the GMT algorithm and therefore both treatment goals will be fully integrated into the GMT algorithm and errorless execution of both complex tasks according to the GMT algorithm will be practiced.

#### GMT-trial and error learning condition

In the conventional GMT errors are allowed to occur. Patients will learn to use the GMT algorithm and the performance of the tasks using trial and error learning. In this condition the therapist is not required to prevent errors during the application of the GMT strategy, but he/she only provides feedback in response to errors (i.e. afterwards). Also, therapists will not provide clues as how to solve problems and will not actively prompt or guide the execution of tasks. After having chosen two (i)ADL tasks in sessions 1 and 2, session 3 consists of a general description of the GMT algorithm by the therapist. In sessions 4 and 5 the participant is asked to define the task steps and- complete the GMT schemes for both treatment goals. The therapist will not help in defining the task steps and the main aim is to motivate the participant to complete the schemes. If errors occur, the therapist will not intervene, and the participant will have to detect these during the training. In sessions 6 and 7 the actual performance of the (i)ADL tasks will be practised, again using the above described trial and error approach. The participant will be motivated to actively practice performance and to seek solutions in the case of problems. Session 8 is again devoted to the execution of the tasks and to eventually improve the execution of task steps or the previous completed GMT schemes.

### Objectives

The primary objective of this investigation is to examine the efficacy of a combined errorless learning and GMT intervention for the treatment of executive problems in patients with acquired brain injury (ABI). These patients are in the chronic phase of their illness and the study will focus on individually chosen complex daily tasks ((i)ADL), such as cleaning a bathroom, processing mail or preparing a meal. The hypothesis is that brain-injured patients will (re)learn performance of (i)ADL tasks more efficiently if an errorless learning method is used. That is, more task steps will be performed correctly and in the right sequence and less irrelevant and missing steps will be present. Consequently, more goals and sub goals will be attained by applying errorless learning in GMT.

### Outcomes

An overview of the outcome measures is given in Table 2. The main outcome will be (i)ADL task performance. Performance of each task step will be scored on a 3-point scale: 0) *absence/incomplete*: the task step is missing or incomplete; 1) *questionable/ineffective*: the task step is not correctly performed or not set in the correct sequence; 2) *competent/correct*: the task step is successfully performed and set in the correct sequence. Observed total scores will be converted into percentage scores to allow statistical comparison of data from different (i)ADL tasks, and comparison between groups. A similar scale was used in previous research [27] to assess (i)ADL task performance in patients with Alzheimer’s dementia. Task execution will be filmed and evaluation by using the scale will take place afterwards by an assessor who is not involved in the actual treatment to secure the blind nature of the design.

**Table 2:** Recruitment- and outcome variables

**Outcome measure**	**Measurement**	**Recruitment**	**Baseline**	**Post-treatment**
**Primary outcome measure**				
*(i)ADL task performance*	Standardized scale evaluating task steps		X	X
***Secondary outcome measure***				
*Goal attainment*	Goal attainment scale		X	X
**Neuropsychological assessment**				
*Executive functioning*	Brixton spatial anticipation test	X		X
	Category fluency test	X		X
	Go/No-go task, subtest TAP	X		X
	Letter fluency test	X		X
	Letter number sequencing, subtest WAIS III	X		X
	Modified six elements test	X		X
	Zoo map test, subtest BADS	X		X
*Memory*	Rivermead behavioural memory test-third edition	X		X
*Attention & Concentration*	Alertness task, subtest TAP	X		X
*Estimation IQ*	National adult reading test (Dutch version)	X		X
**Questionnaires**				
*Subjective cognitive functioning*	CFQ (Cognitive failures questionnaire)		X	X
*Dysexecutive behaviour*	DEX (Dysexecutive questionnaire)		X	X
*Self-reported executive functioning*	EFI-NL (Executive function index)		X	X
*Observed executive functioning*	EOS (Executive observation scale)		X	X
*Quality of life*	RAND 36-item short form health survey		X	X

A secondary outcome measure will be goal attainment using Goal Attainment Scaling (GAS) [46]. GAS is an individualized method to evaluate the extent to which individual treatment goals are achieved by defining several levels of outcomes (‘as expected’, (much) more than expected, (much) less than expected). GAS is scored in a standardized way to allow statistical comparisons between individual treatment goals and is widely used in rehabilitation [47,48]. During session 2, GAS schemes for both treatment goals will be completed by the trainer in cooperation with the participant. During the post-treatment measurement, two GAS scores will be obtained, one by the patient and one by the trainer.

#### Additional study parameters

##### Questionnaires

Questionnaires will be administered to measure several aspects of executive functioning. The Dysexecutive Questionnaire (DEX) [49], both the patient and the proxy version, will be used for the assessment of dysexecutive behaviour. Self-reported executive functioning will be measured using the Dutch version of the Executive Function Index (EFI-NL) [50]. The Executive Observation Scale (EOS) (based on Pollens [51]), completed by a proxy, will be used as an observation measure for executive function. The Cognitive Failures Questionnaire (CFQ) [52] will assess self-reported subjective cognitive complaints in general. Quality of life will be determined using the RAND 36-item Short Form Health Survey (RAND-36) [53].

### Baseline

After the second session, in which two individual treatment goals ((i)ADL tasks) are established and the GAS schemes are completed by the trainer in cooperation with the participant, the baseline measurement will take place. During this assessment execution of both treatment goals will be filmed to secure the blind nature of the design. The recorded performance will be assessed by an independent research assistant using the standardized scale to guarantee blinding of condition.

### Post-treatment

After treatment, (i)ADL task performance will be assessed again by filming and scoring task performance. The previous completed GAS schemes will be scored by participant and by trainer to evaluate goal attainment. The questionnaires and neuropsychological assessment, using parallel versions of the same tests, will be administered after treatment as well, to control for nonspecific recovery. The data gathered with the questionnaires and the neuropsychological tests provide measures for change in insight, executive complaints, subjective and objective executive functioning for moderator analyses, to examine possible determinants for treatment success.

### Sample size

Determination of the sample size for this study is based on data from a RCT examining the effects of a structured 6-week Goal Management Training [17]. In each group 32 participants are required to detect an effect size of *d =* .6 with a power = .80 and α. = 05. These estimated sample sizes are comparable with other studies evaluating the efficacy of different types of GMT [17,54].

### Randomization and blinding

Allocation of participants to either condition will be established using a computer generated block randomization procedure (block size n = 4) without stratification. The written information to inform patients about the study only mentions that two types of GMT will be compared. To achieve participant blinding, no information will be given about specific differences between the two conditions. Assessor blinding will be achieved by filming the (i)ADL task performance of the participants. All hints of treatment condition will be avoided and performance will be scored by research assistants who are not involved in delivering GMT.

### Statistical analysis

All data will be analyzed with IBM SPSS 19. The normality of all variables will be checked and corrected for, if necessary. The performance on the neuropsychological tests will be compared with normative data and corrected for age and education. Descriptive statistics of relevant variables will be obtained and compared for the two treatment arms using analysis of variance.

To evaluate the efficacy of GMT-errorless learning compared to conventional GMT with trial and error learning, pre- and post training data will be analyzed using a 2 × 2 repeated measure analysis of variance (General Linear Model) with treatment condition (GMT-errorless learning and conventional GMT) as between-subject factor and measurement (pre- and post-treatment) as within-subject factor. The dependent variable will be the standardized scale score (quantitative). The same analysis will be done for the secondary outcome measure, the GAS scores. Appropriate post-hoc tests will be performed and effect sizes (partial eta-squared) will be computed. Moreover, correlations will be computed between moderator variables (questionnaires and neuropsychological tests) and treatment effects (difference score: post treatment minus baseline).

The background variables of the participants in both treatment conditions are expected to be comparable (age, education level, estimation IQ) because of the randomization procedure. In case of significant differences, appropriate statistical adjustment for confounding variables will be performed (ANCOVA). All statistical tests will be two-tailed, alpha set at 0.05.

## Discussion

Both Goal Management Training and errorless learning are two methods that have been separately well studied and shown to be effective. Up to now however, the two methods have never been combined. Combining an errorless learning approach with GMT is expected to optimize the acquisition of the GMT algorithm and improve the performance of complex daily tasks in brain-injured patients with executive deficits. Consequently, the efficacy of the intervention is increased, which may contribute to functional independence of patients with acquired brain injury. Not only does the combination of methods provide an evidence-based intervention for clinical practice, the present study may also contribute to more insight into the underlying mechanisms of errorless learning. Previous studies investigating errorless learning have often focussed on patients with profound memory impairments, such as patients with Alzheimer’s disease or Korsakoff’s syndrome [26,30]. The assumption was that due to a dysfunctional explicit memory system errors were not consciously corrected and implicitly consolidated [31]. However, the beneficial effects of errorless learning may also be related to a failing error monitoring system. That is, the inability of patients with executive dysfunction to detect errors [38]. The current study proposal focuses on patients with primarily executive impairments in whom the presence of memory impairments is less prominent. This suggests that explicit memory is relatively spared in these patients, whereas their error-monitoring system is failing. As a result, the current study may contribute to a better understanding of these two underlying mechanisms of errorless learning.

Previous studies evaluated GMT used paper-and-pencil tasks [7] or fixed (i)ADL tasks, such as meal preparation [7] and financial management [15]. A strength of this study is that participants will choose their own individual (i)ADL tasks that will be (re)learned during the training. Individual goals correspond to individual lifestyles and demands and may therefore provide a more fitting contribution to daily functioning and enhance functional independence of the participants.

In summary, the aim of the study is to examine the efficacy of Goal Management Training combined with an errorless learning approach as a treatment of executive problems in patients with acquired brain injury in the chronic phase, focusing on execution of complex daily-life tasks. This study could contribute to a better treatment of executive deficits in cognitive rehabilitation.

### Current study status

The errorless learning in Goal Management Training trial has started recruitment from June 2012.

## Abbreviations

ABI: Acquired brain injury; BADS: Behavioural assessment of the dysexecutive syndrome; CFQ: Cognitive failures questionnaire; CFT: Category fluency test; DEX: Dysexecutive questionnaire; EFI-NL: Executive function index (Dutch version); EOS: Executive observation scale; GAS: Goal attainment scaling; GMT: Goal management training; (i)ADL: (Instrumental) Activities of daily living; IQ: Intelligence quotient; LFT: Letter fluency test; LNS: Letter-number sequencing; NART: National adult reading test; RAND-36: RAND-36 item short form health survey; RBMT III: Rivermead behavioural memory test-third edition; SD: Standard deviation; SPSS: Statistical package for the social sciences; SMART: Specific measurable attainable reasonable timely; TAP: Test for attentional performance; WAIS III: Wechsler adult intelligence scale-third edition

## Competing interests

The authors declare that they have no competing interests.

## Authors’ contributions

DBe is the primary investigator and responsible for writing the treatment protocols, execution of the study, data collection, data analysis and drafting the manuscript. LF and RK are designers and supervisors of the study. DBo contributed to the treatment protocols and is supervising as well. All authors will participate in finalizing the manuscript. All authors read and approved the final manuscript.

## Pre-publication history

The pre-publication history for this paper can be accessed here:

http://www.biomedcentral.com/1471-2377/13/64/prepub
